# Supplementary material for: Wild edible yams from Madagascar: New insights into nutritional composition support their use for food security and conservation
Source: Food Sci Nutr. 2023 Oct 16;12(1):280–91. doi: 10.1002/fsn3.3757 (PMC10804101; doi:10.1002/fsn3.3757)
Supplement: Supplementary file 1 — Table S1 [file FSN3-12-280-s001.docx]

**Supplementary Table 1.** Collection details for edible yam tubers in Madagascar.

| ***Dioscorea* species** | **Harvest month and year** | **Harvest location (GPS)** | **Collection reference codes** |
| --- | --- | --- | --- |
| *D. bako* | August 2018 | Lat 19.9582/ Long 44.6487 | MOR 01  BI 28865 |
| *D. buckleyana* | August 2018 | Lat 12.2125/ Long 49.200 | FEN 409 (M22)  BI 28866 |
| *D. maciba* | August 2018 | Lat 12.2449/ Long 49.2144 | FEN 407 (M42)  BI 28867 |
| *D. orangeana* | August 2018 | Lat 12.1938/ Long 49.2435 | FEN 411 (M52)  BI 28868 |
| *D. pteropoda* | August 2018 | Lat 12.2449/ Long 49.2508 | FEN 406 (M21)  BI 28869 |
| *D. sambiranensis* subsp. *bardotiae* | August 2018 | Lat 12.2117/ Long 49.2023 | FEN 408 (M12)  BI 28870 |
| *D. sambiranensis* subsp. *sambiranensis* | August 2018 | Lat 12.2436/ Long 49.2018 | FEN 413 (M30)  BI 28871 |
| *D. seriflora* | August 2018 | Lat 13.4744/ Long 48.3004 | FEN 445 (M51)  BI 28872 |
| *Dioscorea* species (Ovy valiha) | August 2018 | Lat 12.3729/ Long 49.2447 | FEN 415 (M11)  BI 28873 |
| *D. alata* | October 2018 | Lat 13.4740/ Long 48.2943 | FEN 479 (M33)  BI 29015 |
| *D. bako* | October 2018 | Lat 19.7762/ Long 44.8202 | MOR 02 (M61)  BI 29012 |
| *D. buckleyana* | October 2018 | Lat 12.2125/ Long 49.2001 | FEN 473 (M27)  BI 29107 |
| *D. irodensis* | October 2018 | Lat 12.1836/ Long 49.2245 | FEN 475 (M46)  BI 29008 |
| *D. maciba* | October 2018 | Lat 12.2429/ Long 49.2144 | FEN 471 (M47)  BI 29007 |
| *D. orangeana* | October 2018 | Lat 12.1938/ Long 49.2435 | FEN 474 (M57)  BI 29011 |
| *D. pteropoda* | October 2018 | Lat 12.2449/ Long 49.2508 | FEN 476 (M26)  BI 29013 |
| *D. sambiranensis* subsp. *bardotiae* | October 2018 | Lat 12.2117/ Long 49.2023 | FEN 472 (M17)  BI 29010 |
| *D. sambiranensis* subsp. *sambiranensis* | October 2018 | Lat 13.2842/ Long 48.4528 | FEN 480 (M35)  BI 29009 |
| *D. seriflora* | October 2018 | Lat 12.4744/ Long 49.3004 | FEN 478 (M56)  BI 29016 |
| *Dioscorea* species (Ovy valiha) | October 2018 | Lat 12.3729/ Long 49.2447 | FEN 477 (M16)  BI 29014 |
| *D. alata* | December 2018 | Lat 13.4740/ Long 48.2943 | FEN 521 (M38)  BI 29122 |
| *D. bako* | December 2018 | Lat 19.7762/ Long 44.8202 | MOR 03  BI 29123 |
| *D. buckleyana* | December 2018 | Lat 12.2125/ Long 49.2001 | FEN 505 (M24)  BI 28124 |
| *D. irodensis* | December 2018 | Lat 12.1836/ Long 49.2245 | FEN 507 (M43)  BI 29126 |
| *D. maciba* | December 2018 | Lat 12.2429/ Long 49.2144 | FEN 508 (M44)  BI 29127 |
| *D. orangeana* | December 2018 | Lat 12.1938/ Long 49.2435 | FEN 506 (M54)  BI 299128 |
| *D. pteropoda* | December 2018 | Lat 12.2449/ Long 49.2508 | FEN 509 (M23)  BI 29125 |
| *D. sambiranensis* subsp. *bardotiae* | December 2018 | Lat 12.2117/ Long 49.2023 | FEN 504 (M14)  BI 29129 |
| *D. sambiranensis* subsp. *sambiranensis* | December 2018 | Lat 13.2842/ Long 48.4528 | FEN 522  BI 29130 |
| *D. seriflora* | December 2018 | Lat 12.4744/ Long 49.3004 | FEN 520  BI 29131 |
| *Dioscorea* species (Ovy valiha) | December 2018 | Lat 12.3729/ Long 49.2447 | FEN 510  BI 29132 |
| *D. bako* | March 2019 | Lat 19.7762/ Long 44.8202 | MOR 04  BI 29272 |
| *D. buckleyana* | March 2019 | Lat 12.2125/ Long 49.2001 | GHM 78  BI 29272 |
| *D. irodensis* | March 2019 | Lat 12.1836/ Long 49.2245 | GHM 80  BI 29274 |
| *D. maciba* | March 2019 | Lat 12.2429/ Long 49.2144 | GHM 82  BI 29275 |
| *D. orangeana* | March 2019 | Lat 12.1938/ Long 49.2435 | GHM 79  BI 29276 |
| *D. pteropoda* | March 2019 | Lat 12.2449/ Long 49.2508 | GHM 81  BI 29277 |
| *D. sambiranensis* subsp. *bardotiae* | March 2019 | Lat 12.2117/ Long 49.2023 | GHM 77  BI 29278 |
| *D. sambiranensis* subsp. *sambiranensis* | March 2019 | Lat 13.2842/ Long 48.4528 | GHM 74  BI 29279 |
| *D. seriflora* | March 2019 | Lat 12.4744/ Long 49.3004 | GHM 75  BI 29280 |
| *Dioscorea* species (Ovy valiha) | March 2019 | Lat 12.3729/ Long 49.2447 | GHM 76  BI 29281 |
| *D. alata* | April 2019 | Lat 13.4740/ Long 48.2943 | GHM 122  BI 29753 |
| *D. bako* | April 2019 | Lat 19.9562/ Long 44.65033 | MOR 05  BI 29754 |
| *D. buckleyana* | April 2019 | Lat 12.2125/ Long 49.2001 | GHM 126  BI 29755 |
| *D. irodensis* | April 2019 | Lat 12.1836/ Long 49.2245 | FEN 709  BI 29756 |
| *D. maciba* | April 2019 | Lat 12.2429/ Long 49.2144 | GHM 124  BI 29757 |
| *D. orangeana* | April 2019 | Lat 12.1938/ Long 49.2435 | FEN 708  BI 29758 |
| *D. pteropoda* | April 2019 | Lat 12.2449/ Long 49.2508 | FEN 707  BI 29755 |
| *D. sambiranensis* subsp. *bardotiae* | April 2019 | Lat 12.2117/ Long 49.2023 | GHM 125  BI 29760 |
| *D. sambiranensis* subsp. *sambiranensis* | April 2019 | Lat 13.2842/ Long 48.4528 | FEN 706  BI 29761 |
| *D. seriflora* | April 2019 | Lat 12.4744/ Long 49.3004 | GHM 123  BI 29762 |
| *Dioscorea* species (Ovy valiha) | April 2019 | Lat 12.3729/ Long 49.2447 | FEN 710  BI 29763 |
| *D. alata* | June 2019 | Lat 13.4740/ Long 48.2943 | MIR125  BI 30899 |
| *D. bako* | June 2019 | Lat 19.7762/ Long 44.8202 | MOR 06  BI 30902 |
| *D. buckleyana* | June 2019 | Lat 12.2125/ Long 49.2003 | GHM184  BI 30895 |
| *D. irodensis* | June 2019 | Lat 12.1837/ Long 49.2244 | GHM182  BI 30901 |
| *D. maciba* | June 2019 | Lat 12.2430/ Long 49.2145 | GHM180  BI 30896 |
| *D. orangeana* | June 2019 | Lat 12.1938/ Long 49.2437 | GHM181  BI 30900 |
| *D. pteropoda* | June 2019 | Lat 12.2448/ Long 49.2509 | GHM179  BI 30894 |
| *D. sambiranensis* subsp. *bardotiae* | June 2019 | Lat 12.2118/ Long 49.2024 | GHM183  BI 30897 |
| *D. sambiranensis* subsp. *sambiranensis* | June 2019 | Lat 13.2842/ Long 48.4530 | MIR126  BI 30898 |
| *D. seriflora* | June 2019 | Lat 12.4745/ Long 48.3004 | MIR124  BI 30903 |
| *Dioscorea* species (Ovy valiha) | June 2019 | Lat 12.3728 Long 49.2448 | GHM178  BI 30893 |
| *D. alata* | August 2019 | Lat 13.4740/ Long 48.2943 | GHM188  BI 30910 |
| *D. bako* | August 2019 | Lat 19.7642/ Long 44.6516 | MOR 07  BI 30913 |
| *D. buckleyana* | August 2019 | Lat 12.2005/ Long 49.2228 | MIR151  BI 30906 |
| *D. irodensis* | August 2019 | Lat 12.3422/ Long 49.2436 | GHM185  BI 30912 |
| *D. maciba* | August 2019 | Lat 12.2346/ Long 49.2089 | GHM187  BI 30907 |
| *D. orangeana* | August 2019 | Lat 12.1917/ Long 49.2002 | FEN805  BI 30911 |
| *D. pteropoda* | August 2019 | Lat 12.2448/ Long 49.2508 | GHM186  BI 30905 |
| *D. sambiranensis* subsp. *bardotiae* | August 2019 | Lat 12.2233/ Long 49.1929 | FEN806  BI 30908 |
| *D. sambiranensis* subsp. *sambiranensis* | August 2019 | Lat 13.2843/ Long 48.4526 | MIR152  BI 30909 |
| *D. seriflora* | August 2019 | Lat 13.4745/ Long 48.0841 | FEN807  BI 30914 |
| *Dioscorea* species (Ovy valiha) | August 2019 | Lat 12.3729/ Long 49.2449 | FEN804  BI 30904 |
| *D. alata* | October 2019 | Lat 13.4740/ Long 48.2943 | FEN809  BI 30921 |
| *D. bako* | October 2019 | Lat 19.7642/ Long 44.6516 | MOR 10  BI 30924 |
| *D. buckleyana* | October 2019 | Lat 12.2005/ Long 49.2228 | GHM222  BI 30917 |
| *D. irodensis* | October 2019 | Lat 12.3422/ Long 49.2436 | GHM224  BI 30923 |
| *D. maciba* | October 2019 | Lat 12.2346/ Long 49.2089 | GHM223  BI 30918 |
| *D. orangeana* | October 2019 | Lat 12.1917/ Long 49.2002 | GHM221  BI 30922 |
| *D. pteropoda* | October 2019 | Lat 12.2448/ Long 49.2508 | MIR170  BI 30916 |
| *D. sambiranensis* subsp. *bardotiae* | October 2019 | Lat 12.2233/ Long 49.1929 | FEN810  BI 30919 |
| *D. sambiranensis* subsp. *sambiranensis* | October 2019 | Lat 13.2843/ Long 48.4526 | GHM220  BI 30920 |
| *D. seriflora* | October 2019 | Lat 13.4948/ Long 48.0841 | FEN808  BI 30925 |
| *Dioscorea* species (Ovy valiha) | October 2019 | Lat 12.3729/ Long 49.2449 | FEN811  BI 30915 |
| *D. alata* | December 2019 | Lat 13.4740/ Long 48.2943 | FEN826  BI 31139^a^ / BI 31140^b^ |
| *D. bako* | December 2019 | Lat 19.77318611/ Long 44.83166667 | MOR11  BI 31119^a^ / BI 31120^b^ |
| *D. buckleyana* | December 2019 | Lat 12.2004/ Long 49.2204 | MIR174  BI 31123^a^ / BI 311124^b^ |
| *D. irodensis* | December 2019 | Lat 12.3419/ Long 49.2447 | FEN829  BI 31125^a^ / BI 31126^b^ |
| *D. maciba* | December 2019 | Lat 12.2330/ Long 49.1945 | MIR172  BI 31129^a^ / BI 31130^b^ |
| *D. orangeana* | December 2019 | Lat 12.1917/  Long 49.2001 | MIR175  BI 31121^a^ / BI 31122^b^ |
| *D. pteropoda* | December 2019 | Lat 12.2450/ Long 49.2515 | MIR176  BI 31137^a^ / BI 31138^b^ |
| *D. sambiranensis* subsp. *bardotiae* | December 2019 | Lat 12.2222/ Long 49.1936 | MIR173  BI 31127^a^ / BI 31128^b^ |
| *D. sambiranensis* subsp. *sambiranensis* | December 2019 | Lat 13.2845/ Long 48.4523 | FENj827  BI 31133^a^ / BI 31134^b^ |
| *D. seriflora* | December 2019 | Lat 13.4948/ Long 48.0842 | FEN825  BI 31131^a^ / BI 31132^b^ |
| *Dioscorea* species (Ovy valiha) | December 2019 | Lat 12.3731/ Long 49.2448 | FEN828  BI 31135^a^ / BI 31136^b^ |
| *D. alata* | February 2020 | Lat 13.4740/ Long 48.2943 | GHM226  BI 31159^a^ / BI 31160^b^ |
| *D. bako* | February 2020 | Lat 19.98319765/ Long 44.62176857 | MOR12  BI 31161^a^ / BI 31162^b^ |
| *D. buckleyana* | February 2020 | Lat 12.2004/ Long 49.2204 | GHM227  BI 31143a / BI 31144^b^ |
| *D. irodensis* | February 2020 | Lat 12.3419/ Long 49.2447 | FEN860  BI 31145^a^ / BI 31146b |
| *D. maciba* | February 2020 | Lat 12.2330/ Long 49.1945 | FEN857  BI 31141^a^ / BI 31142^b^ |
| *D. orangeana* | February 2020 | Lat 12.1917/ Long 49.2001 | FEN858  BI 31151^a^ / BI 31152^b^ |
| *D. pteropoda* | February 2020 | Lat 12.2450/ Long 49.2515 | GHM228  BI 31155a / BI 31156^b^ |
| *D. sambiranensis* subsp. *bardotiae* | February 2020 | Lat 12.2222/ Long 49.1936 | FEN859  BI 31149^a^ / BI 31150^b^ |
| *D. sambiranensis* subsp. *sambiranensis* | February 2020 | Lat 13.2845/ Long 48.4523 | FEN856  BI 31147^a^ / BI 31148^b^ |
| *D. seriflora* | February 2020 | Lat 13.4948/ Long 48.0842 | GHM225  BI 31157^a^ / BI 31158^b^ |
| *Dioscorea* species (Ovy valiha) | February 2020 | Lat 12.3731/ Long 49.2448 | FEN861  BI 31153^a^ / BI 31154^b^ |
| *D. alata* | April - May 2020 | Lat 13.474/ Long 48.2943 | GHM225  BI 31248^a^ / BI 31249^b^ |
| *D. bako* | April - May 2020 | Lat 12.2005/ Long 49.2204 | ND  BI 31242^a^ / BI 31243^b^ |
| *D. buckleyana* | April - May 2020 | Lat 12.342/ Long 49.2446 | GHM228  BI 31233^a^ / BI 31234^b^ |
| *D. irodensis* | April - May 2020 | Lat 12.234/ Long 49.1945 | GHM231  BI 31244^a^ / BI 31245^b^ |
| *D. maciba* | April - May 2020 | Lat 12.1917/ Long 49.2002 | GHM233  BI 31240^a^ / BI 31241^b^ |
| *D. orangeana* | April - May 2020 | Lat 12.245/ Long 49.2515 | GHM229  BI 31250^a^ / BI 31251^b^ |
| *D. pteropoda* | April - May 2020 | Lat 12.2224/ Long 49.1936 | GHM230  BI 31238^a^ / BI 31239^b^ |
| *D. sambiranensis* subsp. *bardotiae* | April - May 2020 | Lat 13.2844/ Long 48.4523 | GHM234  BI 31229^a^ / BI 31230^b^ |
| *D. sambiranensis* subsp. *sambiranensis* | April - May 2020 | Lat 13.4948/ Long 48.0843 | GHM226  BI 31252^a^ / BI 31253^b^ |
| *D. seriflora* | April - May 2020 | Lat 12.3731/ Long 49.2448 | GHM227  BI 312486 / BI 31247^b^ |
| *Dioscorea* species (Ovy valiha) | April - May 2020 | Lat 13.474/ Long 48.2943 | GHM232  BI 31235^a^ / BI 31237^b^ |
| *D. alata* | June 2020 | Lat 13.474/ Long 48.2943 | GHM242  BI 31270^a^ / BI 31271^b^ |
| *D. buckleyana* | June 2020 | Lat 12.342/ Long 49.2446 | GHM238  BI 31268^a^ / BI 31269^b^ |
| *D. irodensis* | June 2020 | Lat 12.234/ Long 49.1945 | GHM236  BI 31262^a^ / BI 31263^b^ |
| *D. maciba* | June 2020 | Lat 12.1917 / Long 49.2002 | FEN864  BI 31254^a^ / BI 31255^b^ |
| *D. orangeana* | June 2020 | Lat 12.245/ Long 49.2515 | GHM237  BI 31264^a^ / BI 31265^b^ |
| *D. pteropoda* | June 2020 | Lat 12.2224/ Long 49.1936 | GHM240  BI 31266^a^ / BI 31267^b^ |
| *D. sambiranensis* subsp. *bardotiae* | June 2020 | Lat 13.2844/ Long 48.4523 | GHM239  BI 31272^a^ / BI 31273^b^ |
| *D. sambiranensis* subsp. *sambiranensis* | June 2020 | Lat 13.4948/ Long 48.0843 | MIR131  BI 31258^a^ / BI 31259^b^ |
| *D. seriflora* | June 2020 | Lat 12.3731/ Long 49.2448 | GHM241  BI 31256^a^ / BI 31257^b^ |
| *Dioscorea* species (Ovy valiha) | June 2020 | Lat 13.474 / Long 48.2943 | GHM235  BI 31260^a^ / BI 31261^b^ |

^a^Parenchyma; ^b^Periderm.

Samples were harvested at two-monthly periods except for collections in March and April 2019 (due to adverse weather conditions) and in May and June 2020 (due to a lockdown during the covid-19 pandemic). Samples were air-dried at room temperature in the shade for 10-20 days (depending on the species), except for samples harvested in December 2018, which were dried in an oven at 65°C for 2 days.
